# Electroretinographic evaluations of retinal function before, just after, and after intravitreal injections

**DOI:** 10.1038/srep31104

**Published:** 2016-08-05

**Authors:** Kazuma Yagura, Kei Shinoda, Soiti Matsumoto, Gaku Terauchi, Makoto Kawashima, Emiko Watanabe, Harue Matsumoto, Takeshi Iwata, Atsushi Mizota, Yozo Miyake

**Affiliations:** 1 Department of Ophthalmology, Teikyo University School of Medicine, 2-11-1, Kaga, Itabashi-ku, Tokyo 173-8605, Japan; 2 Matsumoto Eye Clinic, Tokushima, Japan; 3 Division of Molecular and Cellular Biology, National Institute of Sensory Organs, National Hospital Organization Tokyo Medical Center, Tokyo, Japan; 4 Aichi Medical University, 1-1 Yazakokarimata, Nagakute, Aichi, 480-1195, Japan

## Abstract

Intravitreal injections (IVI) have become a part of daily practice for a growing number of procedures. We evaluated the retinal function by recording intraoperative photopic electroretinograms (ERGs) before an injection (T1), just after the injection (T2), and after the aspiration of the anterior chamber fluid (T3) of 19 eyes of 19 patients (mean age 70.6 years; men = 11) who received an IVI of an anti-vascular endothelial growth factor. The mean amplitudes of the b-wave, photopic negative responses (PhNR), and oscillatory potentials (OPs) 1 and 2 at T2 were significantly smaller than that at T1, but no significant difference was observed between T3 and T1. The mean implicit times of the a-wave and OP1, 2, and 3 at T2 and the a-wave and the OP2 at T3 were significantly longer than that at T1. The mean intraocular pressure (IOP) at T2 (49.32 mm Hg) was significantly higher and the IOP at T3 (8.74 mm Hg) was significantly lower than that at T1 (21.05 mm Hg). The retinal function was reduced and the IOP elevated just after the IVI. The response of each ERG component was different suggesting a different sensitivity of each type of retinal neuron to IVI.

An intravitreal injection of anti-vascular endothelial growth factor (anti-VEGF) agents has become a common procedure for several types of retinal diseases, e.g., exudative age-related macular degeneration (AMD), macular edema associated with retinal vein occlusion (RVO), diabetic retinopathy, and other retinal diseases associated with vascular abnormalities^1^,^2^,^3^,^4^,^5^,^6^,^7^,^8^. In addition, the number of intravitreal injections of ocriplasmin has increased worldwide^9^,^10^,^11^. Thus, intravitreal injections have become a part of the daily practice for a growing number of procedures.

The adverse effects of intravitreal injections include endophthalmitis, cataract progression, vitreous hemorrhage, and retinal tears^1^,^2^,^12^,^13^. A transient elevation of the intraocular pressure (IOP) is known to occur immediately after an intravitreal injection and the elevation of the IOP may be sustained^14^,^15^,^16^. An elevated IOP is an important risk factor for glaucoma, which raises a concern about the long-term safety of intravitreal injections^17^,^18^ especially in eyes with risk factors for ocular hypertension and/or glaucoma. However, no information about the effects of intravitreal injections on retinal function in humans has been published.

Miyake and colleagues^19^,^20^,^21^ recorded intraoperative electroretinograms (ERGs) during vitreous surgery and reported a reduction in the amplitude and prolongation of the implicit time of the 30 Hz flicker ERGs. However, an accurate evaluation of each type of retinal cells was not performed, and measurements of the IOP were not made.

Thus, the purpose of this study was to determine whether the retinal function is altered during and after an intravitreal injection of anti-VEGF drugs. In addition, the effect of the intravitreal injection on the IOP was determined. To accomplish this, we recorded photopic ERGs and measured the IOPs before and just after the intravitreal injection. In addition, ERGs were recorded after the IOP was lowered by anterior chamber (AC) paracentesis^22^,^23^,^24^. The photopic ERGs allowed us to do detailed analyses of the function of the cone pathway, and we were able to evaluate the changes in the cone-driven retinal function before, during, and after the IVI.

## Patients and Methods

### Patients

The participants were scheduled to undergo an intravitreal injection of an anti-VEGF antibody for different reasons at the Teikyo University Hospital in Tokyo, Japan in 2015. All of the patients gave an informed consent for the operation with intraoperative ERG recordings and IOP measurements. Patients with severe high myopia (>−6.0 D or axial length >26 mm) and/or glaucoma were excluded to minimize the effect of more vulnerable retinas.

We studied 11 eyes of 11 men and 8 eyes of 8 women. The average age of the patients was 70.6 ± 13.7 years (±SD) with a range from 35 to 87 years. The vitreoretinal pathologies were; 8 with exudative AMD, 3 with macular edema due to branch RVO, 2 with central RVO, and 7 with diabetic macular edema (DME). The number of previous IVI received by the patients varied from 0 to 16 with a mean of 3.7 ± 1.0, mean ± SD). Eight eyes received ranibizumab and 11 eyes received aflibercept.

This study was conducted according to the guidelines of the Declaration of Helsinki, and all of the procedures were approved by the Ethics Committee of the Teikyo University School of Medicine. An informed consent was obtained from all subjects.

## Methods

The procedures were performed in accordance with the approved guidelines. All of the intravitreal injections were performed under topical anesthesia by 4% lidocaine. Patients were prepped and draped in the usual sterile fashion, and after sterilization of the ocular surface with povidone iodine, either ranibizumab (0.5 mg/0.05 ml) or aflibercept (2.0 mg/0.05 ml) was injected into the vitreous cavity through the pars plana using a 30-gauge needle. After the injection, a paracentesis was performed to normalize the IOP. The room temperature was set at 25.0 degree centigrade throughout the operation.

Intraoperative ERGs (iERGs) were recorded using a contact lens with a built-in light-emitting diode (LS-100, Mayo Co, Inazawa, Japan) according to the method reported by Miyake *et al.* (1991, Arch Ophthalmol). ERGs were recorded before the injection (T1), just after the injection (T2), and after the aspiration of the anterior chamber fluid (T3). The IOP was recorded just before each ERG recording with the Tono-pen AVIA (Reichert, USA).

### Intraoperative electroretinograms (iERGs)

A contact lens with a built-in light-emitting diode (LS-100, Mayo Co, Inazawa, Japan) was sterilized and used as both the stimulus source and the recording electrode for the photopic iERGs. The reference electrode was a silver plate placed on the forehead and the ground electrode was attached to one ear lobe. The photopic ERGs were elicited by 2-Hz rectangular stimuli of 3-ms duration and a luminance of 1000 cd/m^2^. The luminance of the constant background was 25.1 cd/m^2^.

The iERGs were amplified, A/D converted, and averaged with a bioamplifier (MEB-9404, Nihon Kohden Corporation, Tokyo, Japan). Twenty responses were averaged at a sampling rate of 10 kHz. The responses were filtered with a hardwired band pass filter between 2 Hz to 1 kHz to record the a- and b -waves and between 100 Hz to 500 Hz to record the oscillatory potentials (OPs). The analysis time was 300 ms. All ERG recordings were performed after 5 minutes of light-adaptation to the room light.

**Figure 1:**
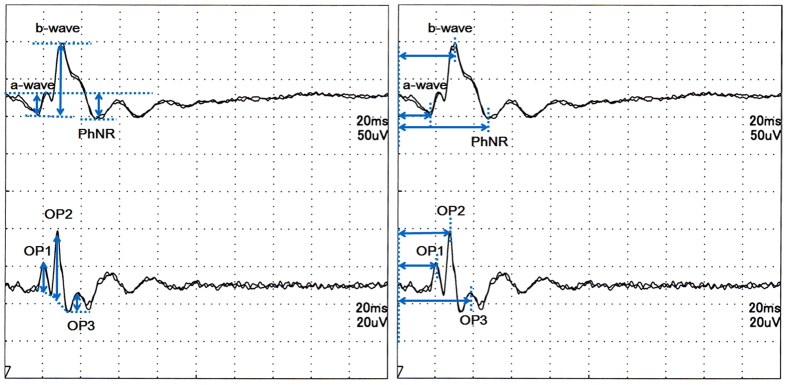
Measurements of the amplitude and implicit time of each component. Left: The amplitude of the a-wave was measured from the baseline to the trough of the a-wave, the amplitude of the b-wave was measured from the trough of the a-wave to the peak of the b-wave, and the amplitude of the photopic negative response (PhNR) was measured from the baseline to the trough of the PhNR. The amplitude of the OPs was measured from the baseline which was determined as the line connecting each negative trough of the OPs to the peak. Right: The implicit times of the a-, and b-waves, the PhNR, and the OPs were measured from stimulus onset to the peak of each wave.

The amplitudes and implicit times of the a- and b-waves, photopic negative responses (PhNRs) following the b-wave, and OPs were analyzed. Briefly, the implicit times of the a-, and b-waves, the PhNR, and the OPs were measured from stimulus onset to the peak of each wave (Fig. 1). The amplitudes of the a-waves were measured from baseline to the troughs of the a-waves, and the amplitudes of the b-waves were measured from the troughs of the a-waves to the peaks of the b-waves. The amplitudes of the PhNR were measured from the baseline to the trough following the b-wave (Fig. 1). The amplitudes of the OPs were measured from the baseline, which was defined as the line connecting each negative trough to the peak (Fig. 1).

### Test-re-test reproducibility

To examine the reproducibility of the ERG recordings, two recordings were recorded and evaluated. The intraclass correlation coefficients (ICC [1,^2^]) were calculated for average amplitude and implicit time for each component at T1, where the ERG recordings were done twice before the intervention. The time elapsed between the two measurements was approximately one minute. According to Fleiss, ICCs ≥0.75, between 0.40 and 0.75, and ≤0.4, are defined as excellent, moderate, and poor, respectively^25^.

### Statistical analyses

Two-way analysis-of-variance (ANOVA; three time points vs. ERG components) was used to test for the significance of the procedural effects, and the null hypothesis was that there were no effects of the procedures. Post hoc tests were performed at the three time points. Paired *t* tests were used to determine the significance in the differences of the values for each of the six ERG components independently. The significance of differences in the IOPs, amplitudes, and implicit times of the a-, b-, and d-waves, PhNR, and OPs, between T1 and T2 and between T1 and T3 was determined by paired *t* tests. The α-level was set to 0.006 to compensate for the multiple comparisons. The *t* tests for the amplitude on the a-wave, b-wave, and PhNR were done for T1 vs T2, T2 vs T3, and T1 vs T3. The number of comparisons was 9 and the α-level was set at 0.05 divided by 9 thereby 0.006. The *t* tests for the amplitudes of OP1, OP2, and OP3 were done in the same way and the α-level was set at 0.006. The α-level for the implicit time was set at 0.006 in the same way. Pearson’s correlation coefficient was used to determine the significance of the correlations between the changes of the IOP and each ERG parameter. Because IOPs >55 mmHg and <5 mmHg was not designated by the Tono-pen AVIA, the reading of ‘Over’ was assigned a value of 56 mmHg and ‘Under’ was assigned a value of 4 mmHg for the statistical analyses. Spearman correlation coefficients were calculated to determine the correlations between the number of previous IVIs and the changes in the amplitudes and the implicit times of each component.

### Conformity to Ethical Requirements

The procedures used conformed to the tenets of the Declaration of Helsinki. The study was a observational case series with approval of the Ethics Committee of the Teikyo University School of Medicine (Study ID Number: 10-033-2) and informed consent was obtained from all participants to participate in research.

## Results

The intraobserver reproducibility of the ERG measurements was excellent. The ICC [1, 2] was 1.000, 0.997, 0.988, 1.000, 0.999, and 0.992 for the amplitude of a-wave, b-wave, PhNR, OP1, OP2, and OP3, respectively. It was 1.000, for the implicit time of all components. The correlations between the number of previous IVIs and the changes in the amplitudes and the implicit times of each component were not significant.

**Figure 2:**
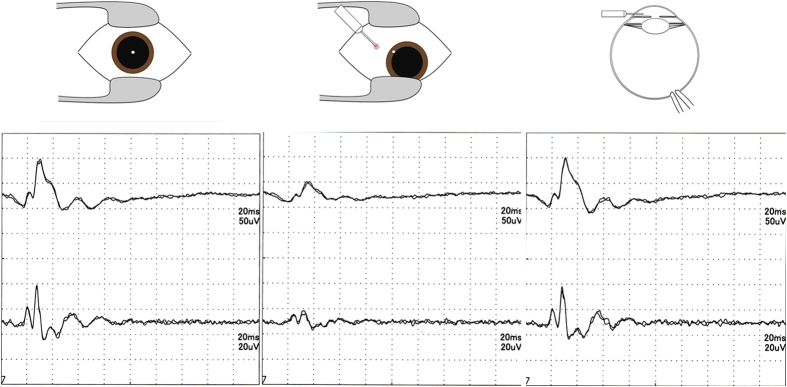
Electroretinograms (ERGs) in a representative case. Left: ERG before intravitreal injection at T1. The intraocular pressure (IOP) was 18 mmHg. Middle: The ERG just after the intravitreal injection at T2. The IOP was 41 mmHg. Right: The ERG just after aspiration of anterior chamber fluid at T3. The IOP was 9 mmHg.

**Figure 3:**
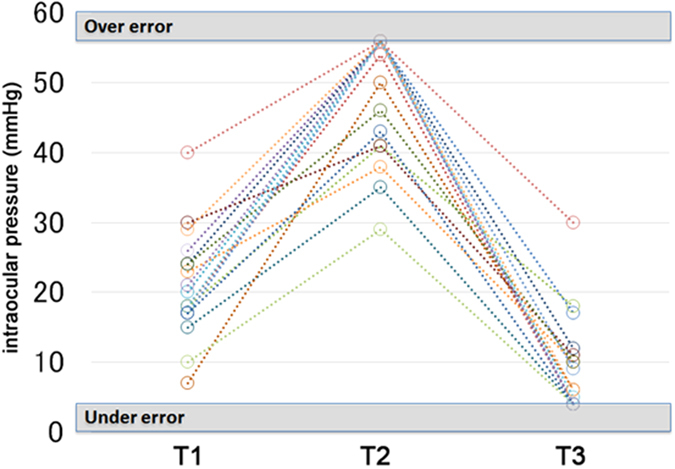
Changes of the intraocular pressure in all eyes. The intraocular pressures (IOPs) are plotted for each eye at the three time points. When the scale reads ‘over error’ as shown, the IOP is assigned as 56 mmHg, and when the scale reads ‘under error’, the IOP is assigned as 4 mmHg. The IOP of 10 eyes were assigned to be 56 mmHg at T2 and 8 eyes were assigned as 4 mmHg at T3. T1, time before the injection; T2, time immediately after the injection; and T3, time after the paracenthesis.

Representative ERGs are shown in Fig. 2. The IOP of each eye at three time points is shown in Fig. 3. Among the 57 measurements, 18 had the out-range reading; 10 eyes had an ‘over error’ at T2 and 8 eyes had an ‘under error’ at T3.

Two-way ANOVA showed that the ERG parameters were not consistent at the three time points *(P < *0.001 for both amplitude and implicit time). Scheffe’s F tests showed that the amplitude was significantly different between T1 and T2 (*P* = 0.004) and T2 and T3 (*P* = 0.024), and the implicit time was significantly different between T1 and T2 (*P* = 0.003).

**Figure 4:**
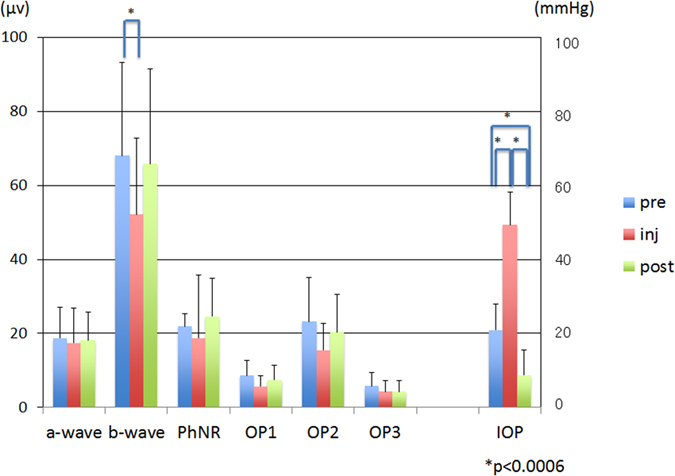
The mean amplitude of each component of the ERGs and the mean intraocular pressure (IOP) at each time. The mean amplitude of the b-wave at T2 was significantly smaller than that at T1. No significant difference in the mean amplitude of each component was observed between T1 and T3. The mean IOP at T2 was significantly higher than that at T1, and it at T3 was significantly lower than that at T1. PhNR, photopic negative response; OP, oscillatory potentials; pre at T1, inj at T2, post at T3. The error bars represent the standard deviations. The asterisks indicate that the differences were significant at, * = *P* < 0.006.

**Figure 5:**
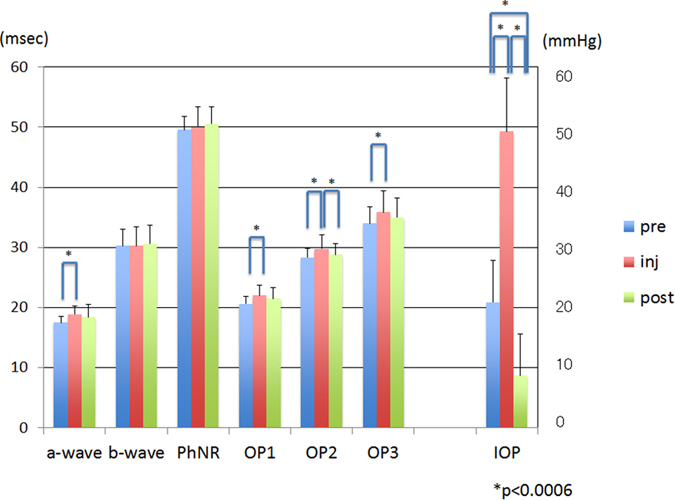
The mean implicit time of each component of the ERG and the mean intraocular pressure (IOP) at each time (T). The mean implicit time of each component except the b-wave and the PhNR at T2 is significantly longer than at T1, and the mean implicit time of the OP2 at T3 was significantly shorter than that at T2. PhNR, photopic negative response; OP, oscillatory potentials; before = T1; immediately after the injection = T2; and immediately after the paracentesis = T3. The error bars represent the standard deviations. The asterisks indicate that the differences were significant at, * = *P* < 0.006.

The mean amplitude of the b-wave at T2 was significantly smaller than that at T1 (*P* < 0.006, Fig. 4, Table 1). However, the mean amplitude of each component at T1 vs T3 and at T2 vs T3 was not significantly different (*P* > 0.006, Fig. 4, Table 1). Similarly, the mean implicit time of each component except the b-wave and the PhNR at T2, was significantly longer than that at T1 (*P* < 0.006, Fig. 5, Table 1). However, the differences of the mean implicit times of each component between T1 vs T3 and T2 vs T3 were not significant (*P* > 0.006, Fig. 5, Table 1).

Additional comparisons were made between the parameters that showed significant changes from T1 to T2, and no significant difference was found in the changes in the implicit times for the a-wave, OP1, OP2, and OP3.

The mean IOP was 49.32 ± 8.83 mm Hg at T2 which was significantly higher than at T1 (21.05 ± 7.48 mm Hg, *P* < 0.001). The IOP recovered to 8.74 ± 6.81 mmHg at T3, which was significantly lower than that at T1 and T2 (*P* < 0.001, Figs 4 and 5, Table 1).

**Figure 6:**
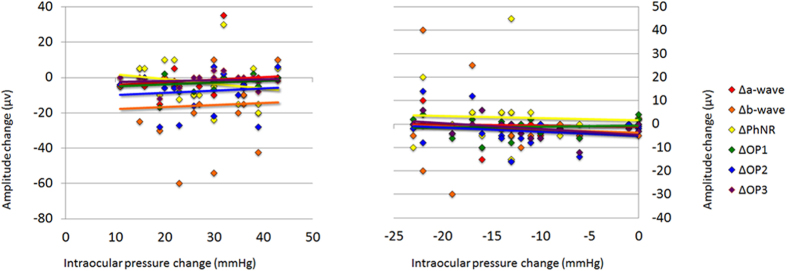
Relationship between the amplitude changes and the intraocular pressure (IOP) changes. The coefficients of correlation between the differences in the amplitudes at T1 and T2, i.e., T2 minus T1 (Δamp), for the different components and the difference in the IOP at T1 and T2, i.e., T2 minus T1 (ΔIOP), are not significant (Fig. 6A). The coefficients of correlation between the differences in the amplitudes at T1 and T3, i.e., T3 minus T1 (ΔIOP), for the different components and the differences in the IOPs at T1 and T3, i.e., T3 minus T1 (ΔIOP) are also not significant (Fig. 6B). Note that for the ΔIOP calculations, when the scale reads ‘over error’ as shown, the IOP was assigned a value of 56 mmHg, and when the scale reads ‘under error’, the IOP was assigned a value of 4 mmHg. PhNR: photopic negative response, OP: oscillatory potentials.

The coefficients of correlation between the differences in the amplitudes (Δamp) at T1 and T2, i.e., T2 minus T1, of each component and the differences of the IOPs at T1 and T2, i.e., T2 minus T1 (ΔIOP), were not significant (*P* > 0.05, Fig. 6). Similarly, the coefficients of correlation between the differences in the amplitudes at T1 and T3, i.e., T3 minus T1 (ΔIOP), of each component and the differences in the IOPs at T1 and T3, i.e., T3 minus T1 (ΔIOP) were not significant (*P* > 0.05, Fig. 6). Although there was a tendency for the amplitudes of the b-waves, the PhNRs, and the OP2 to be smaller with the higher IOPs at T1 to T2, the differences were not statistically significant.

**Figure 7:**
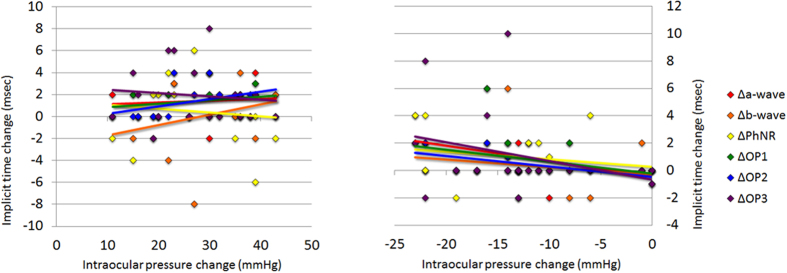
Relationship between the implicit time change and the intraocular pressure (IOP) change. The coefficients of correlation between the change in the implicit times at T1 and T2, i.e., T2 minus T1 (Δimp), and the change in the IOPs at T1 and T2, i.e., at T2 minus T1 (ΔIOP), are not significant (Fig. 7A). The relationship between the change of the implicit times from T1 to T3 and the change of the IOPs from T1 to T3, i.e., T3 minus T1 (ΔIOP), are significantly correlated with the change of the OP2 from T1 to T3 (Fig. 7B). Note that for the ΔIOP calculations, when the scale reads ‘over error’ as shown, the IOP was assigned a value of 56 mmHg, and when the scale reads ‘under error’, the IOP was assigned a value of 4 mmHg. PhNR: photopic negative response, OP: oscillatory potentials.

The coefficients of correlation between the differences in the implicit times at T1 and T2, i.e., T2 minus T1 (Δimp), for each component, and the differences in the IOPs at T1 and T2, i.e., T2 minus T1 (ΔIOP) were not significant (*P* > 0.05, Fig. 7). There was a tendency for the implicit times of the b-waves, the OP1s, and OP2s to be more prolonged with increasing IOPs from T1 to T2, but the differences were not statistically significant. However, the relationship between the differences in the implicit times of the OP2 from T1 to the T3, T3 minus T1 (Δimp), and the differences of the IOPs from T1 to T3, i.e., T3 minus T1 (ΔIOP), were significantly correlated (*P* < 0.05, Fig. 7). The implicit time of OP2 was more prolonged with a decrease in the IOP from T1 to T3. Although the difference was not significant, there was a similar tendency in the a-wave, the PhNR, the OP1, and the OP3.

## Discussion

Electrophysiological evaluations of retinal function during eye surgery was first reported by Miyake *et al.*^19^,^20^,^21^. They recorded flicker ERGs and reported significant changes in retinal function during vitreous surgery and scleral buckling surgery^19^,^20^,^21^. We recorded photopic iERGs and IOPs before, just after the intravitreal injections of anti-VEGF drugs, and after AC paracentesis. The photopic ERGs arise from the electrical activities of the cones and inner layer retinal cells that are elicited by light stimuli. The a-wave of the photopic ERGs results from a combination of the electrical activity from the cone photoreceptors and the OFF bipolar cells elicited at the onset of the light stimuli. The b-waves arise mainly from the on-pathway bipolar cells and Müller cells, and the PhNR arises from the electrical activity of the retinal ganglion cells. Photopic ERGs were used to evaluate the functional status of the outer and inner retinal cells of the cone pathway.

Our measurements showed that the retinal function was significantly decreased just after the intravitreal injections in conjunction with significant elevations of the IOP. The ERG components partially recovered to the baseline level just after the aspiration of the anterior chamber fluid which led to a decrease in the IOP. The reduction of the IOP by AC paracentesis (measure T3) did not significantly increase the ERG amplitude but decreased the implicit time. The explanation may be that the T3 measurement values were between the T1 and T2 values, i.e., a partial return to normal. The partial recovery resulted in T3 values that were not different statistically from either of the other two measures.

Thus, the changes in the iERGs are likely related to the IOP increase, but other confounding factors might also be involved, e.g., temperature changes in the vitreous cavity, the effects of the contents of the injected drugs, and condition of the optic nerve. In terms of the intravitreal temperature, a slightly decrease in the temperature probably occurred after the injection. However, measurements of the intraocular temperature with an intravitreal probe would be invasive and was not done. Because the amount of the anti-VEGF agent injected was relative small compared to the volume of the vitreous cavity, the change in the intraocular temperature was small, and its influence on the ERG responses was most likely limited.

Several investigators have reported that the IOP can increase to 40 to 100 mmHg during different types of eye surgery, e.g., vitrectomy, scleral indentation, phacoemulsification, femtosecond laser-assisted cataract surgery, and laser *in situ* keratomileusis^26^,^27^,^28^,^29^,^30^,^31^,^32^,^33^. The increase in the IOP is important because it has been reported that the stress on the posterior chamber-anterior hyaloid membrane increases as the IOP increases^34^. In addition, an increase in the IOP above the systolic arterial pressure can damage the retina and other ocular tissues^35^. Even with increases in the IOP to lower levels, retinal ganglion cell damage can be induced in association with an elevation of the extracellular ATP by the abrupt IOP elevation^36^. In addition, there is evidence that wide variations in the IOPs may be directly related to an increase in the postoperative inflammatory processes^37^.

The depression of the retinal function in conjunction with the IOP elevation was most likely due to an impairment of ocular circulation. Yancey *et al.*^38^ investigated the effect of IOP elevation on the full-field ERGs and the choroidal oxygen tension (PO_2_) under dark-adapted conditions in feline eyes. They reported that the choroidal PO_2_ and b-wave amplitudes were decreased with increases in the IOP. Tazawa *et al.*^39^ recorded full-field ERGs from living extracorporeal bovine eyes, and reported that the b-wave was the first to be decreased, and the a-waves were later decreased under anoxic conditions. With re-perfusion, the a-waves were the first to recover followed by the b-waves. Vadala *et al.*^40^ reported that the photopic OPs were altered by IOP elevations. The current results are in good agreement with these results.

Animal experiments^41^,^42^,^43^ have shown that the scotopic threshold responses (STR) and OPs, which originate from the activity of the neural cells in the inner retinal layer, were more sensitive to acute IOP elevation than the cells contributing to the a- and b-waves. Chrysostomou *et al.*^44^ reported that the PhNR was reduced by an acute elevation of the IOP. The PhNR and the STR are believed to reflect the activity of the inner retinal layer especially the retinal ganglion cells, and our data partly agree with the results of the earlier animal data in that significant alterations in the OPs from T1 to T2 were observed.

Although these changes may be transient and generally do not cause severe and permanent adverse changes, we should remember that the number of intravitreal injections is rapidly increasing, and it may be performed repeatedly on the same eye. Intravitreal injection-associated visual depression has been reported^45^,^46^. Although the correlations between the number of previous IVIs and the changes in the ERG parameters were not significant, a possibility that repeated intravitreal injections might lead to permanent reductions should be considered especially in eyes with risk factors such as glaucoma, ocular hypertension, uveitis, and optic neuropathy. Recently, Hahn *et al.* reported that prior intravitreal anti-VEGF injections may be a risk factor for cataract surgery-related intraoperative complications and endophthalmitis^47^. Our results highlight the clinical impact of a single intravitreal injection on the retinal function that has not been considered.

Interestingly, the changes were different for the different components of the iERGs. The differences in the degree of alterations of each ERG component suggests different sensitivities of each type of retinal neuron. Some ERG components may respond differently depending on the disease process. For example, the OPs are attenuated in the early stage of diabetic retinopathy^48^ because the retinal ganglion cells are susceptible to ischemia^49^,^50^, and the PhNR may be more altered in eyes with retinal circulatory disorders^51^. The ERG changes may also be related to other unidentified factors rather than just by the IOP. The intravitreal injections may cause changes in the electrolytes and osmolality as well as the temperature which may contribute to the ERG changes^52^,^53^,^54^.

The changes in the ERGs and IOPs were not significantly correlated although there was a tendency that the higher IOP elevations were associated with a greater decrease in some of the components when the T1 and T2 times were compared. These findings should be interpreted cautiously because IOPs over 55 mmHg and under 5 mmHg could not be measured. In addition, the ERG recordings and IOP measurements were not made simultaneously. Therefore, the IOP might be changed during the ERG recordings. The lack of the significant correlation between the ERG and IOP may be attributed to these factors.

Interestingly, the implicit time of the OP2 was more prolonged with a decrease in the IOP from T1 to T3. In addition, there was a similar tendency for the a-waves, the PhNRs, the OP1s, and the OP3s. These findings contradict the observations by Niyadurupola *et al.*^55^ who reported that a decrease in the IOP was associated with an increase in the amplitude of the PhNR in eyes with glaucoma and hypertension. Our observation on the prolongation of the implicit time at T3 cannot be compared simply in these studies because it was after T2 when the implicit time had already increased together with the temporal increase of the IOP. Further studies where the IOP is directly reduced from baseline will be helpful to clarify the ERG response during simple IOP lowering.

Our study has several limitations. One is that the retinal pathology of the patients was different. A second limitation is that the tolerance of the retinal function in specific retinal disease against the IOP changes is not well understood. A larger number of patients would enable subgroup analysis with each vitreoretinal disease and then ERG changes during intravitreal injections for specific vitreoretinal diseases can be determined. In addition, the IOP values had a limited range. Taking these limitations into consideration, we still believe that the current data are clinically important.

In conclusion, the results show that the retinal function is reduced and the IOP is elevated just after the intravitreal injection of anti-VEGF drugs. The response of each ERG component to the injections varied suggesting different sensitivities of each type of retinal neuron to the rise in the IOP.

## Additional Information

**How to cite this article**: Yagura, K. *et al.* Electroretinographic evaluations of retinal function before, just after, and after intravitreal injections. *Sci. Rep.* **6**, 31104; doi: 10.1038/srep31104 (2016).

## Figures and Tables

**Table 1:** Changes of the electrophysiologic parameters and the intraocular pressure at three time points.

amplitude (uV)									
a-wave		*P* value		b-wave	*P* value		PhNR	*P* value	
T1	18.68 ± 8.30	T1 vs T2	0.58278	68.03 ± 25.23	T1 vs T2	***0.0026***	21.84 ± 3.45	T1 vs T2	0.30478
T2	17.37 ± 9.48	T1 vs T3	0.66288	52.16 ± 20.59	T1 vs T3	0.54666	18.74 ± 17.00	T1 vs T3	0.34477
T3	18.16 ± 7.63	T2 vs T3	0.74158	65.92 ± 25.59	T2 vs T3	0.01058	24.61 ± 10.35	T2 vs T3	0.12321
implicit time (msec)									
a-wave		*P* value		b-wave	*P* value		PhNR	*P* value	
T1	17.47 ± 1.07	T1 vs T2	***0.00166***	30.26 ± 2.75	T1 vs T2	1	49.57 ± 2.19	T1 vs T2	0.51974
T2	18.89 ± 1.37	T1 vs T3	0.03763	30.26 ± 3.19	T1 vs T3	0.39983	50 ± 3.42	T1 vs T3	0.03218
T3	18.42 ± 2.04	T2 vs T3	0.32435	30.63 ± 3.06	T2 vs T3	0.66689	50.53 ± 2.91	T2 vs T3	0.24204
amplitude (uV)									
OP1		*P* value		OP2	*P* value		OP3	*P* value	
T1	8.63 ± 4.06	T1 vs T2	0.01184	23.21 ± 12.00	T1 vs T2	0.00713	5.74 ± 3.65	T1 vs T2	0.09272
T2	5.68 ± 2.89	T1 vs T3	0.20152	15.47 ± 7.24	T1 vs T3	0.09894	4.21 ± 2.99	T1 vs T3	0.13104
T3	7.42 ± 3.99	T2 vs T3	0.13693	20.37 ± 10.15	T2 vs T3	0.05522	4.26 ± 3.01	T2 vs T3	0.94804
implicit time (msec)									
OP1		*P* value		OP2	*P* value		OP3	*P* value	
T1	20.63 ± 1.16	T1 vs T2	***0.0003***	28.32 ± 1.53	T1 vs T2	***0.00036***	34 ± 2.75	T1 vs T2	***0.00555***
T2	22.05 ± 1.65	T1 vs T3	0.02811	29.79 ± 2.3	T1 vs T3	0.02451	35.89 ± 3.56	T1 vs T3	0.17793
T3	21.47 ± 1.87	T2 vs T3	0.14975	28.79 ± 1.84	T2 vs T3	***0.00193***	35 ± 3.28	T2 vs T3	0.10472
intraocular pressure									
		*P* value							
T1	20.9 ± 6.93	T1 vs T2	***1.1E-10***						
T2	49.32 ± 8.83	T1 vs T3	***4.6E-07***						
T3	8.73 ± 6.81	T2 vs T3	***2.2E-12***						

T1: time point 1, before injection, T2: time point 2, just after injection.

T3: time point 3, just after aqueous humor aspiration.

PhNR:photopic negative response, OP:oscillatory potential.

*P*-value of significance is shown in italic.
